# Juglone induces apoptosis of tumor stem-like cells through ROS-p38 pathway in glioblastoma

**DOI:** 10.1186/s12883-017-0843-0

**Published:** 2017-04-07

**Authors:** Jinfeng Wu, Haibo Zhang, Yang Xu, Jingwen Zhang, Wei Zhu, Yi Zhang, Liang Chen, Wei Hua, Ying Mao

**Affiliations:** 1 Department of Dermatology, Huashan Hospital, Fudan University, #12 Middle Wurumuqi Road, Shanghai, 200040 People’s Republic of China; 2 Department of Neurosurgery, Huashan Hospital, Fudan University, #12 Middle Wurumuqi Road, Shanghai, 200040 People’s Republic of China; 3 Institutes of Biomedical Sciences, Fudan University, #131 Dong’an Road, Shanghai, 200040 People’s Republic of China; 4 Department of Ultrasound, Hebei General Hospital, #348 West Heping Road, Shijiazhuang, Hebei Province 050000 People’s Republic of China; 5 State Key Laboratory of Medical Neurobiology, School of Basic Medical Sciences and Institutes of Brain Science, Fudan University, Shanghai, 200040 People’s Republic of China; 6 The Collaborative Innovation Center for Brain Science, Fudan University, Shanghai, 200040 People’s Republic of China

**Keywords:** Juglone, Glioma, Tumor stem-like cells, Apoptosis, Reactive oxygen species

## Abstract

**Background:**

Juglone is a natural pigment, which has cytotoxic effect against various human tumor cells. However, its cytotoxicity to glioma cells, especially to tumor stem-like cells (TSCs) has not been demonstrated.

**Methods:**

TSCs of glioma were enriched from U87 and two primary cells (SHG62, and SHG66) using serum-free medium supplemented with growth factors, including bFGF, EGF and B27. After treatment of juglone with gradient concentrations (0, 10, 20, and 40 μM), the viability and apoptosis of TSCs were evaluated by WST-8 assay and flow cytometry. Reactive oxygen species (ROS) was labeled by the cell-permeable fluorescent probe and detected with flow cytometry. ROS scavenger (NAC) and p38-MAPK inhibitor (SB203580) were applied to resist the cytotoxic effect. Caspase 9 cleavage and p38 phosphorylation (P-p38) were quantified by western blot. Juglone as well as temozolomide (TMZ) were administrated in intracranial xenografts and MR scan was performed every week to evaluate the anti-tumor effect in vivo.

**Results:**

Juglone could obviously inhibit the proliferation of TSCs in glioma by decreasing cell viability (*P* < 0.01) and inducing apoptosis (*P* < 0.01), which was accompanied by increased caspase 9 cleavage in a dose-dependent manner (*P* < 0.01). In the meantime, juglone could generate ROS significantly and increase p38 phosphorylation (*P* < 0.01). In addition, pretreatment with ROS scavenger or p38-MAPK inhibitor could reverse juglone-induced cytotoxicity (*P* < 0.01). More importantly, juglone could also suppress tumor growth in vivo and improve the survival of U87-bearing mice compared with control (*P* < 0.05), although TMZ seemed to have better effect.

**Conclusions:**

Juglone could inhibit the growth of TSCs in gliomas through the activation of ROS-p38-MAPK pathway in vitro, and the anti-glioma effect was validated in vivo, which offers a potential therapeutic agent to gliomas.

## Background

As one of the most deadly primary brain tumors, glioblastoma (GBM) has the characteristics of rapid growth and high invasiveness. The median survival time of GBM has been prolonged to about 14.6 months even after comprehensive treatments of surgery, chemotherapy and radiotherapy [1]. After many endeavors, temozolomide (TMZ) emerged as a feasible first-line chemotherapeutic agent through DNA alkylation in glioma cells, which was validated by phase III clinical trial [2, 3]. However, some tumors without MGMT methylation have been reported to be resistant to TMZ, and thus limiting its efficiency [4]. In the meantime, a subgroup of quiescent tumor stem-like cells (TSC) have been demonstrated to re-initiate tumor growth after TMZ treatment [5]. Bevacizumab (anti-VEGF﻿A﻿), which could only benefit proneural subtype of GBM [6], has also not been encouraging. Therefore, it is necessary to find some novel chemotherapeutic agents targeting GBM.

Natural products have recently received much attention as potential therapeutic agents, e.g., matrine as a cell cycle blocker [7], camptothecin as a proliferation inhibitor [8], and podophyllotoxin as an apoptosis inducer [9]. Similarly, juglone, a lipid-soluble drug, has been widely used as a chemotherapeutic agent in Chinese herbal medicine against various tumors, including leukaemia [10], melanoma [11], gastric cancer [12] and pancreatic cancer [13] through the activation of apoptotic caspase cascade and the increase of ROS (reactive oxygen species) [14, 15]. Recently, juglone has been found to inhibit cell proliferation and to reduce the invasiveness of C6 rat glioma cells in vitro [16]. However, if it could exert a cytotoxic effect in vivo remains unknown.

TSCs in glioma could not be completely eliminated even through combined treatment modality, and thus become the main reason of chemotherapy resistance and the root of tumor relapse [17]. Therefore, we explored the anti-tumor effect of juglone to glioma TSCs and its potential mechanism in this study. Furthermore, we also compared its effect with TMZ in order to provide an available alternative for patients after chemotherapeutic treatment failure.

## Methods

### Glioma stem-like cells culture

U87 was purchased from American Type Culture Collection (Manassas, VA). GBM primary cells (SHG62 and SHG66) were established in our laboratory previously [18]. The glioma TSCs were cultured in serum-free medium (DMEM/F12) supplemented with growth factors, including 10 ng/mL bFGF (basic Fibroblast Growth Factor), 20 ng/mL EGF (Epidermal Growth Factor), and B-27 (1:50 dilution; Life Technologies, Carlsbad). Cell cultures were maintained in a 5% CO_2_ humidified incubator at 37 °C.

### Cell viability assays

Juglone (St Louis, MO) was dissolved in dimethyl sulfoxide (DMSO) and diluted in DMEM/F12. The final working concentration of DMSO was 100 mM. Cell viability was measured by the WST-8 assay (Kumamoto, Japan) following optimized manufacturer’s recommendation. Briefly, cells were seeded at a density of 2 × 10^4^cells/200ul/well in 96-well plates, and then incubated overnight in serum-free medium. The cells were pretreated with and without NAC (a ROS scavenger, 2 mM), or SB203580 (an inhibitor of p38-MAP kinase, 5 μM) for 1 h. Then the cells were treated with different concentrations of juglone (0, 10, 20, and 40 μM). After 48 h incubation, 20 μl WST-8 was added to each well, and the cells were incubated for another 6 h. The optical density (OD) was detected at 450 nm with microplate spectrophotometer (BD Biosciences, San Jose, CA). The percentage of viable cells was determined by the formula: ratio (%) = [OD (juglone) - OD (blank)/OD (control) -OD (blank)] × 100. The experiment were triplicated, and each contained six replicates.

### Cell apoptosis and death assay

For cell apoptosis assay, GBM cells in serum-free medium were treated with juglone (0, 20, and 40 μM) for 48 h, 1 × 10^5^ cells were harvested and incubated in 100 μL labeling solution (5 μL of Annexin V FITC, 5 μL of PI, 10 μL of 10 × binding buffer and 80 μL of H_2_O) in darkness at room temperature for 15 min, after that, 400 μL of binding buffer was added to stop the staining reaction. For cell death assay, the cells were pretreated with or without NAC (Sigma Aldrich, 2 mM), or SB203580 (Sigma Aldrich, 5 μM) for 1 h. Then the cells were treated with juglone (0, 40 μM) for 48 h. Following incubation, cells were collected and fixed in 70% ethanol for 24 h at 4 °C. After that, the cells were resuspended in 500 μL phosphate buffer solution (PBS) containing RNaseA (10 mg/mL, 50 μL) and PI (2 mg/mL, 10 μL). The mixture was incubated in the dark at 37 °C for 30 min. For cell apoptosis and death assay, cells were then analyzed on a FACS Calibur cytometer (Becton Dickinson, San Joe CA). The data were analyzed using FlowJo software V6.0 (Tree star, Ashland OR). Early apoptotic cells are defined as annexin V^+^/PI^−^, whereas late apoptotic/necrotic cells are defined as annexin V^+^/PI^+^. The extent of cell death was determined by evaluating the sub G1 fraction. The experiments were triplicated.

### Evaluation of ROS generation

ROS was labeled by the cell-permeable fluorescent probe (2^,^,7^,^- Dichloro- fluorescein diacetate, DCFDA, Sigma Aldrich) and detected with flow cytometry. Briefly, cells were exposed to various concentrations of juglone (0, 20, and 40 μM) for 24 h and then loaded with DCFDA (10 μM) in serum-free medium. Following incubation at 37 °C for 30 min, cells were washed with PBS and fluorescence was measured with flow cytometry. The mean fluorescence intensity (MFI) data was analyzed by FlowJo software. The MFI experiments were repeated three times.

### Western blot assay

Cells were treated with different concentrations of juglone (0, 20, and 40 μM) for 48 h. Total protein extracts were obtained from lysis buffer (150 mM NaCL, 1% NP-40, 0.5% sodium deoxycholate, 0.1% SDS, and 50 mM Tris-Cl pH 8.0, 2 ug/mL aprotinin, 2 ug/mL leupeptin, 40 mg/mL of phenylmethylsulfonyl fluoride, 2 mM DTT). The protein concentration was determined by the Bradford assay (BioRad, Hercules, CA), and samples were separated on SDS-PAGE, and then transferred onto polyvinylidene difluoride (PVDF) membranes. The membranes were immunoblotted with primary Abs against cleaved caspase 9 (Cell Signaling Technology, 1:1000), P-p38 (Cell Signaling Technology,1:1000), and β-actin (Cell Signaling Technology,1:10000) overnight at 4 °C, followed by horseradish peroxidase (HRP) conjugated secondary Ab (BioRad,1:3000). Detection was carried out using Supersignal West Femto Chemiluminescent Substrate (Pierce, Rockford, IL). β-actin was taken as reference and the band intensities were quantified using UN-SCAN-IT gel analysis software (Silk Scientific, Orem, UT).

### Cytotoxicity of juglone on glioma stem-like cells in vivo

Female BALB/c-nu mice (8–10 week) were obtained from SlacLaboratoryAnimal Company (Shanghai, China). Animal experiment was conducted according to protocols approved by the Institutional Animal Care and Use Committee at Fudan University. Animals were housed with a 12 h light/dark cycle, and acclimated to their environment as least 1 week prior to experimentation. Micewere anesthetized intraperitoneally with 10% chloral hydrate and stereotactically inoculated with 1 × 10^5^ U87 stem-like cells in 10uL PBS *via* micro-syringe into the right forebrain (2.5 mm lateral and 1 mm anterior to bregma, at a 3 mm depth from the skull surface). 3 days after the inoculation, the mice were randomly distributed into three groups, including vehicle control group, juglone treatment group, and TMZ treatment group. The number of animals in each group was 8. Juglone or TMZ was dissolved in DMSO and diluted in PBS; the final concentration of DMSO was 20 mg/ml [19]. PBS containing the same concentration of DMSO was used as vehicle control. Juglone treatment group was injected intraperitoneally with juglone (1 mg/kg) every 3 days, while TMZ treatment group was injected intraperitoneally with TMZ (25 mg/kg) every day. The total drug injections were 5 times per animal. The mice were monitored every three days,and the tumor were evaluated weekly using enhanced MR scan (1.5 T, gadolinium,Bayer Schering Pharma AG,0.2 ml/kg).

### Statistics

All data were presented as the mean ± standard deviation (SD). Data analysis was performed by one-way analysis of variance (*ANOVA*). For comparison of two groups, a student’s *t*-test was used. Differences with *P* values < 0.05 were considered to be statistically significant.

## Results

### Juglone is cytotoxic to glioma stem-like cells

**Fig. 1:**
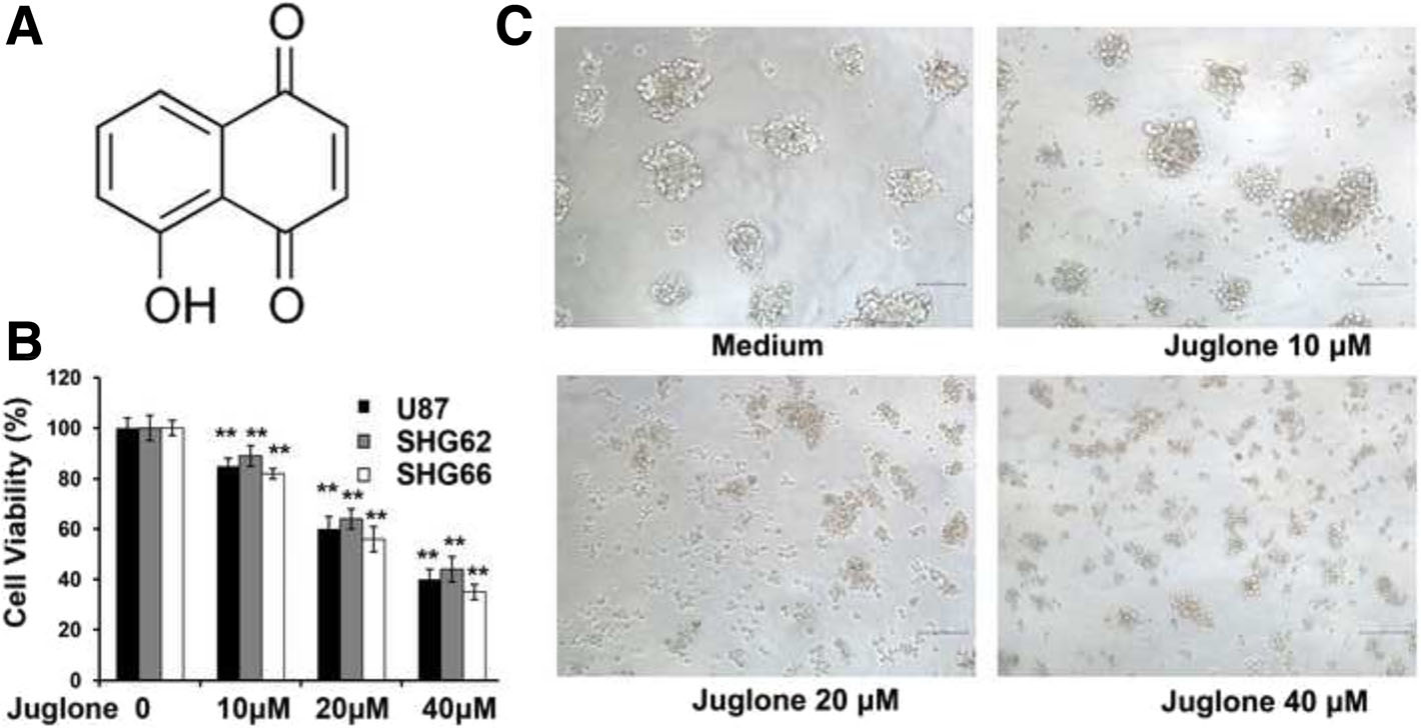
Juglone could decrease cell viabilityof glioma stem- like cells in vitro. **a** Chemical structure of juglone. **b** Cell viability (U87, SHG62 and SHG66) decreased obviously after treatment with juglone at various concentrations (10, 20, and 40 μM) as compared to control (**P < 0.01). **c** The cell morphology (100×) of juglone-treated U87 showed that stem-like cell spheres formation decreased gradually after juglone treatment in a dose-dependent manner

The stem-like cell viability of U87, SHG62 and SHG66 were evaluated by WST-8 assay after treatment with juglone for 48 h. As shown in Fig. 1b, juglone (10, 20, 40 μM) could dramatically decrease the viability of glioma stem-like cells as compared to the control (*P* < 0.01), and the cell viability fell significantly to 65.3 ± 5.06% and 40.7 ± 8.21% after treated with higher concentration of juglone. Meanwhile, stem-like cell spheres formation decreased gradually after juglone treatment in a dose-dependent manner, accompanied with cell shrinkage, reduction of cell adherence (Fig. 1c).

### Juglone could induce glioma stem-like cells apoptosis

**Fig. 2:**
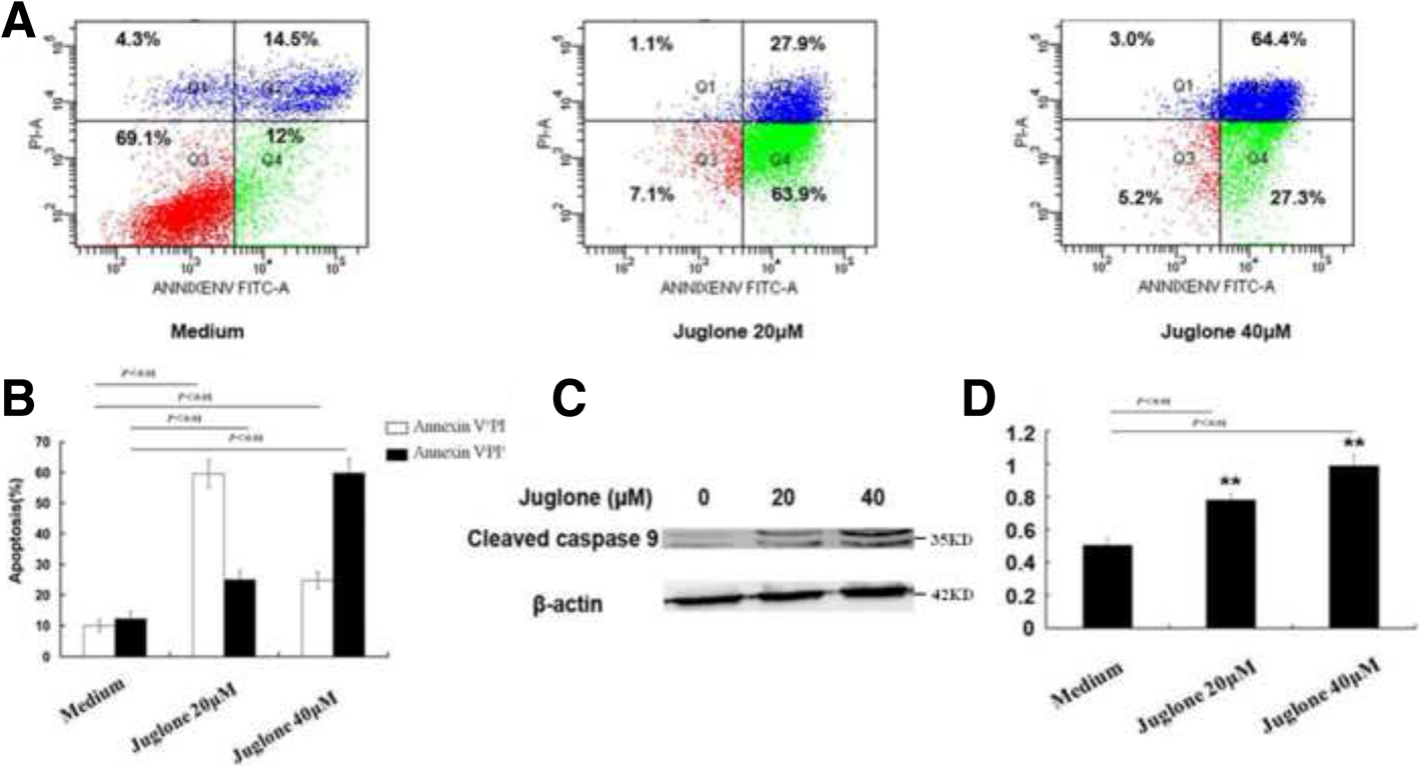
Juglone could induce apoptosis of glioma stem-like cells. **a** FACS analysis indicated that juglone could significantly induce apoptosis at different concentrations. **b** Statistic analysis indicated that juglone could significantly induce both early apoptosis and late apoptosis as compared to the control. **c** Juglone increased caspase 9 cleavage. **d** Quantification of caspase 9 cleavage with western blot (**P < 0.01)

To determine if juglone could induce apoptosis, the percentage of Annexin V^+^PI^−^ and Annexin V^−^PI^+^ U87 stem-like cells after concentration gradients (0, 20, and 40 μM) were measured with flow cytometry. As shown in Fig. 2a and b, juglone (20 μM) increased percentage of Annexin V^+^PI^−^and Annexin V^−^PI^+^cells by 49.1 ± 9.15% and 11.1 ± 7.15% as compared to control (*P* < 0.01). However, juglone (40 μM) increased the percentage of Annexin V^+^PI^−^ and Annexin V^−^PI^+^ cells by 12.1 ± 7.35% and 51.1 ± 8.52% as compared to control (*P* < 0.01). In the meantime, juglone could increase caspase 9 cleavage (Fig. 2c and d), which indicated juglone-induced apoptosis.

### Juglone could generate ROS and activate p38-MAPK pathway

**Fig. 3:**
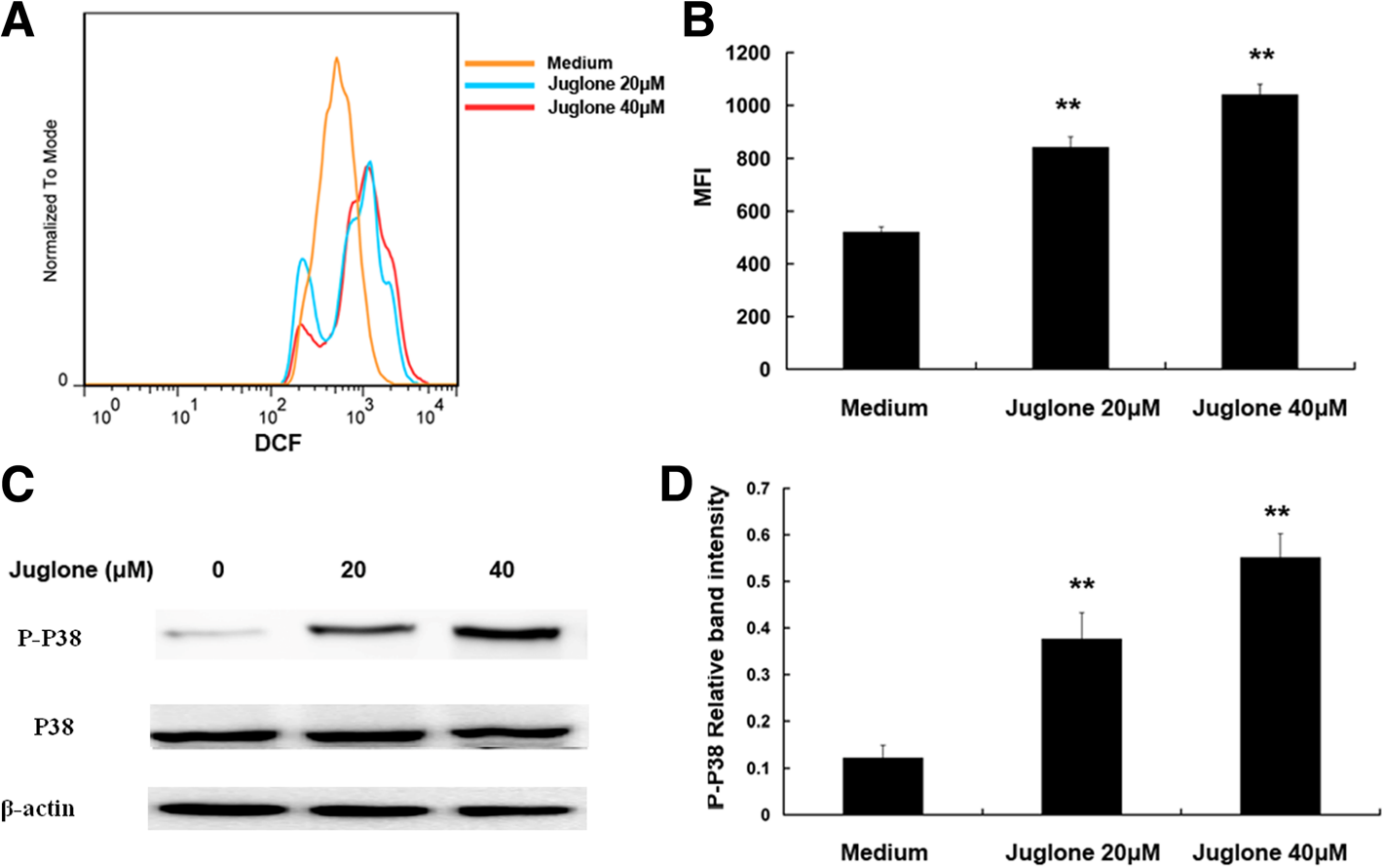
Juglone could generate ROS and activate p38 phosphorylation. **a** Flow cytometry showed that juglone-induced ROS generation was increased in a dose-dependent manner. **b** Statistical data of ROS MFI in different groups (**P < 0.01). **c** Juglone treatment significantly increased p38 phosphorylation in a dose-dependent manner. **d** Statistical data of P-p38 protein at different concentrations using western blot (**P < 0.01)

Juglone-induced ROS generation was obvious in a dose-dependent manneras compared to vehicle control (*P* < 0.01) (Fig. 3a and b). As demonstrated by western blot (Fig. 3c and d), juglone (20, 40 μM) treatment significantly increased p38 phosphorylation (*P* < 0.01),which indicated p38-MAPK pathway activation in TSCs.

### NAC and SB203580 pretreatment could reverse juglone-induced growth inhibition of glioma stem-like cells

**Fig. 4:**
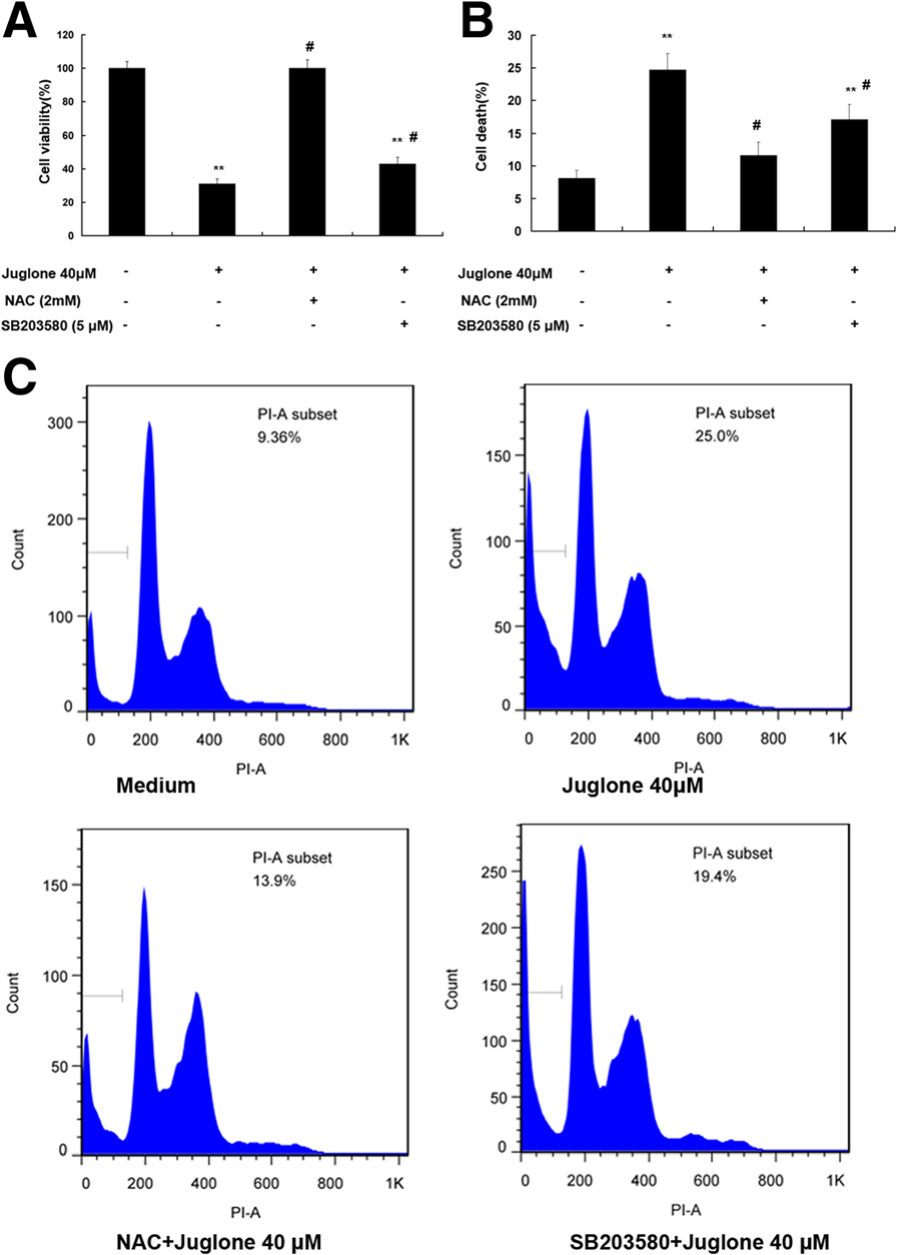
Pretreatment with NAC and SB203580 could reverse juglone-induced inhibition of glioma stem-like cells. **a** Statistic analysis indicated that NAC or SB203580 could resist juglone-induced cell viability decrease (**P < 0.01). **b** Statistic analysis indicated that NAC or SB203580 could resist juglone-induced cell death (**P < 0.01). **c** Flow cytometry showed that NAC or SB203580 pretreatment could reverse the decrease of cell viability and the increase of cell death induced by juglone (**P < 0.01)

As demonstrated by WST-8 assay (Fig. 4a), juglone (40 μM) treatment could decreased cell viability by 65 ± 2.53% (*P* < 0.01), whereas NAC and SB203580 could reversed the cytotoxic effect of juglone by 75 ± 3.18% (*P* < 0.01) and 58 ± 3.92%(*P* < 0.01). Cell death data (Fig. 4b and c) showed that treatment with juglone (40 μM) increased cell death by 17 ± 3.87% (*P* < 0.01), while NAC and SB203580 pretreatment could reverse juglone-mediated increases of cell death by 12.4 ± 2.33% (*P* < 0.01) and 7.1 ± 2.91% (*P* < 0.01). All those results indicated that ROS-p38-MAPK pathway was involved in the juglone-induced cytotoxicity.

### Juglone could reduce glioma growth in vivo and improve the survival of glioma-bearing mice

**Fig. 5:**
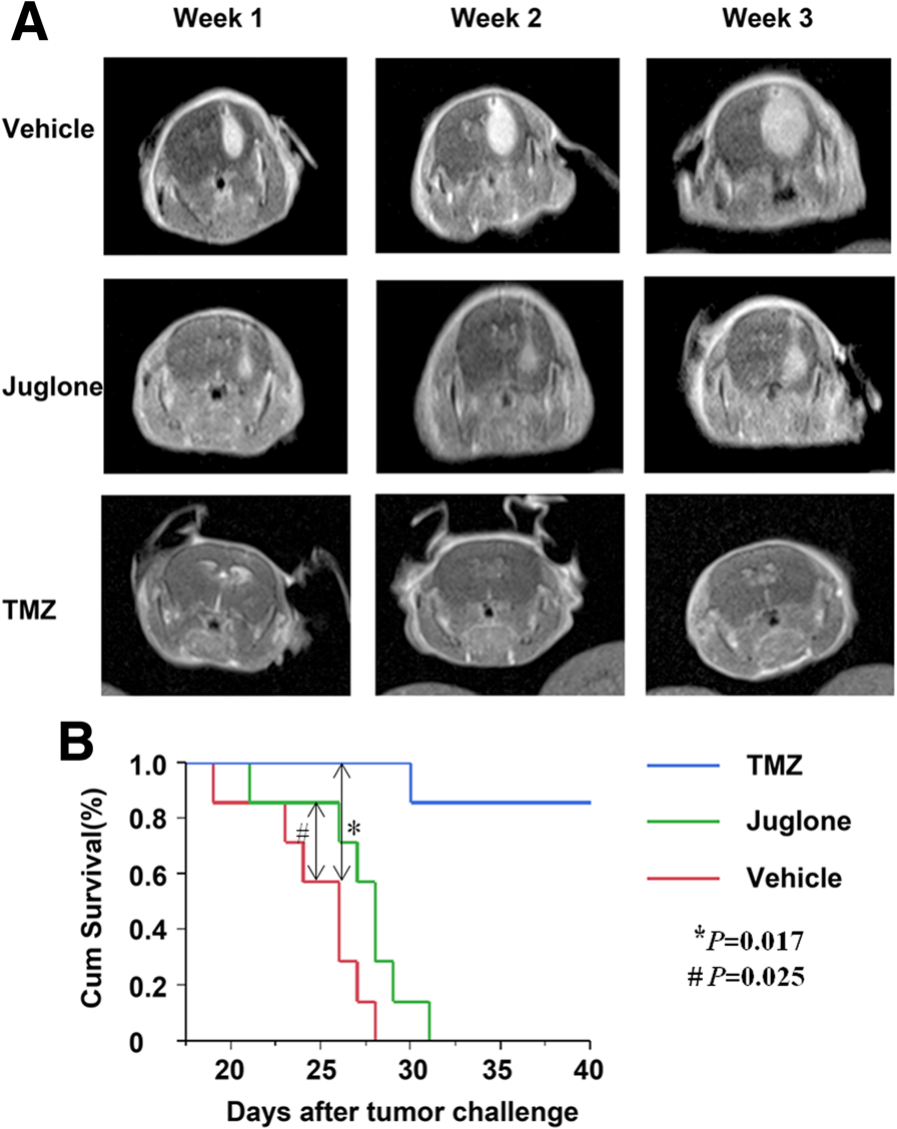
Juglone could retard glioma growth in vivo and prolong the survival time of glioma-bearing mice. **a** MR images showed that juglone group (1 mg/kg), as TMZ group (25 mg/kg) (*P* = 0.017),could also retarded glioma growth in vivo comparing with control group (*P* = 0.025). **b** Juglone treatment could improve, though less significant than TMZ, the survival status of glioma-bearing miceas compared with control (*P* < 0.05)

Brain tumor models were successfully established, and the anti-glioma effect of juglone in vivo was investigated. Both juglone and TMZ could markedly retard glioma growth in vivo confirmed by the MR scan results (Fig. 5a). In the meantime, juglone and TMZ could both significantly increase the survival time of glioma-bearing mice as compared to control (*P* = 0.025, *P* = 0.017, respectively. Figure 5b) and juglone could increase the survival time by about 23.6%. Obviously, TMZ had a better cytotoxic effect than juglone in vivo. All these results demonstrated that juglone could be an effective anti-glioma agent.

## Discussion

Since TSCs are responsible for resistance to chemotherapy [5], novel therapeutic strategies targeting specifically to TSCs are urgently needed. Spectrum Collection Library (MicroSource, Gaylordsville, CT) was designed to screen small compounds for anti-tumor chemotherapeutic agents, and obtusaquinone (one natural product) was identified to have pro-apoptotic effect on TSCs in vitro and to suppress tumor in vivo [20]. However, the suppression ratio was not as high as expected, and many new promising agents needed confirmation by clinical studies. Here, we reported a natural pigment-juglone, a lipid-soluble drug, which could easily pass the blood brain barrier and exert anti-tumor effect against GBM cells, especially against TSCs in vitro and in vivo.

In current study, juglone treatment could inhibit TSCs growth by inducing apoptosis. We also observed that juglone treatment could increase caspase 9 cleavage which was consistent with previous study [14]. Juglone treatment could induce apoptosis of glioma cells at both early and late stage. These results indicated that juglone could exert different biological function under different concentrations.

Many possible mechanisms have been reported to be involved in the juglone-induced anti-tumor effect, among which ROS-based pathways were investiagted the most [10, 12]. Tumor cells presented higher level of ROS than normal cells [21]. In addition, high ROS concentration could induce cell apoptosis and necrosis in relation with the severity and the duration of exposure [22]. Therefore, many ROS-inducing agents are currently used in clinical trials for different tumors [21, 23]. These agents could not only act as direct inhibitors of cancers, but also sensitize tumor cells to chemotherapies [24]. In this study, we evaluated the growth inhibition and apoptosis induction by juglone in human GBM TSCs in vitro and in vivo, and we also confirmed that ROS was involved in juglone-induced apoptosis. These findings were further supported by the pretreatment with NAC, which could block the cytotoxicity of juglone as a ROS scavenger. Previous studies indicated that ROS could induce the activation of the p38-MAPK pathway, which is involved with apoptosis [25, 26]. In this study, we also found that juglone could activate the p38-MAPK pathway *via* ROS generation, and pretreatment with SB203580 could reverse the ROS-induced effect. Besides p38-MAPK pathway, many other pathways could be activated by ROS, such as ROS-AMPK-mTOR pathway and ROS-ERK/AKT-p53 pathway [27, 28], which need to be validated in gliomas. Juglone could also exert cytotoxic effect as a Pin-1 (Peptidyl-prolyl cis/trans isomerase 1) inhibitor through caspase cascade in nasopharyngeal carcinoma [29], which need further research. Therefore, juglone might inhibit TSCs through multiple mechanisms.

There were also some limitations in this study. Juglone could exert better anti-glioma effect than TMZ in vitro (data not shown). However, the cytotoxic effect of juglone was poorer than that of TMZ in vivo, which is partially due to the various anti-tumor mechanisms. In the meantime, the dosage of juglone was low (1 mg/kg) in animal experiment due to the consideration of the side effects. So, we can modify specific chemical groups to reduce its side effects while maintaining its cytotoxicity. The elaboration of anti-tumor mechanism by juglone and better understanding of TSCs would also contribute to the future treatment of gliomas. At least, juglone could offer us another alternative for GBM patients with TMZ resistance or failure, which needs further clinical investigation.

## Conclusions

Juglone could inhibit proliferation and induce apoptosis of glioma stem-like cells in vitro and in vivo, which was mediated through the activation of ROS-p38- MAPK pathway. So, juglone might serve as a potential chemotherapeutic agent for gliomas.

## Abbreviations

GBM: Glioblastoma
MFI: Mean fluorescence intensity.
ROS: Reactive oxygen species
TMZ: Temozolomide
TSCs: Tumor stem-like cells
